# Determination of Blast Vibration Safety Criteria for Buried Polyethylene Pipelines Adjacent to Blast Areas, Using Vibration Velocity and Strain Data

**DOI:** 10.3390/s23146359

**Published:** 2023-07-13

**Authors:** Shengwu Tu, Dongwang Zhong, Linna Li, Xiangchao Gong, Haohao Tao

**Affiliations:** College of Science, Wuhan University of Science and Technology, Wuhan 430065, China; tsw0936@wust.edu.cn (S.T.); lilinna@wust.edu.cn (L.L.); gongxiangchao@wust.edu.cn (X.G.); taohaohao@wust.edu.cn (H.T.)

**Keywords:** polyethylene pipes, blast vibration sensors, model tests, safe vibration velocity criteria

## Abstract

In order to ensure the safe operation of buried polyethylene pipelines adjacent to blasting excavations, controlling the effects of blasting vibration loads on the pipelines is a key concern. Model tests on buried polyethylene pipelines under blasting loads were designed and implemented, the vibration velocity and dynamic strain response of the pipelines were obtained using a TC-4850 blast vibrometer and a UT-3408 dynamic strain tester, and the distribution characteristics of blast vibration velocity and dynamic strain were analyzed based on the experimental data. The results show that the blast load has the greatest effect on the circumferential strain of the polyethylene pipe, and the dynamic strain response is greatest at the section of the pipe nearest to the blast source. Pipe peak vibration velocity (PPVV), ground peak particle velocity (GPPV), and the peak dynamic strain of the pipe were highly positively correlated, which verifies the feasibility of using GPPV to characterize pipeline vibration and strain level. According to the failure criteria and relevant codes, combined with the analysis of experimental results, the safety threshold of additional circumferential stress on the pipeline is 1.52 MPa, and the safety control vibration speed of the ground surface is 21.6 cm/s.

## 1. Introduction

In recent years, with the continuous advancement of urban infrastructure construction, buried pipeline projects are intertwined and the environment is complex and changeable. Polyethylene material is widely used in underground pipeline gas transportation and water supply and drainage projects, due to its high strength, high temperature resistance, corrosion resistance, non-toxicity, wear resistance, and lower cost than ordinary iron pipes and steel pipes. At the same time, with the rapid development of urban transportation, a large number of underground construction projects, such as subways, have sprung up. These projects often run parallel to, intersect, and span existing underground pipelines. Hard rocks are often encountered in the excavation process of underground engineering. As an efficient excavation method, blasting has been widely used, but blasting construction often affects adjacent pipelines. Therefore, it is of engineering practical significance and theoretical research value to study the dynamic response of buried polyethylene pipelines under the action of blasting loads and analyze the controlled vibration velocity of the pipelines.

Currently, a large number of scholars have carried out research work on the effects of blast vibration loads on adjacent pipelines [1,2,3,4]. In terms of studying the deformation characteristics of pipelines using the method of indoor tests, Ha et al. [5] used centrifuge tests to study the deformation law of HDPE pipelines, and combined the stress–strain data to obtain the relationship between the lateral force and deformation of the pipeline. Abdoun et al. [6] used centrifuge tests to study the force performance of PE pipelines with different burial depths and pipe diameters. Wang et al. [7] conducted indoor similar model tests to study the vibration characteristics of rock and adjacent buried pipes, and the dynamic response law of pipes during the construction of underpass tunnel by drill-and-blast method. In addition, a large number of scholars have used numerical simulation to study the dynamic response of buried pipes [8,9,10]. Francini et al. [11] used blasting numerical calculations to study the vibration law of the adjacent buried pipes and the ground surface above them, and proposed corresponding safety criteria. Jiang et al. [12] used the field monitoring data of Beijing metro line 16 to establish a 3D numerical model, analyzed the effects of subway tunnel blasting, and studied the dynamic response characteristics of the pipeline and the surrounding soil. Zhang Zhen et al. [13] established numerical models by LS-DYNA through field inspection, and studied the vibration velocity of buried concrete pipe sections at different locations under the action of shallow bursting vibration. Zhu [14] proposed the dynamic response of a ductile iron gas pipeline under blasting vibration by a field test. Xia [15] studied the dynamic response characteristics of a socket concrete pipeline through field tests, and put forward the vibration velocity safety criterion of the pipeline. Zhong [16] obtained the dynamic response of a polyethylene pipeline under explosive load by using field model tests. At present, studies on the dynamic response of adjacent pre-buried pipes under blast vibration conditions mostly use numerical software and indoor model tests [17,18,19,20], and few studies have been conducted using field tests. However, the numerical model and indoor model tests deal with the internal conditions and the external environment of the pipeline in a simple way, which makes a big difference compared with the actual engineering situation. Further, most of the research objects are hard pipes, such as cast iron and concrete, but there are fewer studies on polyethylene pipes, which are widely used but have a soft texture.

In this study, blasting tests on buried polyethylene pipes were first designed and implemented, using a TC-4850 blast vibrometer and UT3408 dynamic strain tester to establish a monitoring system to test and record the vibration velocity and dynamic strain of the pipes under blast vibration loads, respectively. The test data was then used to analyze the response characteristics of the polyethylene pipes under blast vibration loads at different explosive loads. Finally, using the pipe stress yielding criterion, the safety performance of the pipeline was evaluated based on the additional annular stress and the ground safety control vibration velocity of the polyethylene pipeline in conjunction with the relevant codes. The results show that this approach is feasible and effective.

## 2. The Experimental Setup

### 2.1. Experimental Site Parameters and Piping Parameters

The test site is located in an empty site of a blasting test center. The soil in the site has a high water content, and the soil medium is mainly yellow clay. The water content and saturation of the soil increase with the soil depth, and the saturation is between 60% and 100%; at about 2.5 m deep, the soil is silt-like, and the soil parameters vary greatly at different depths. The SR-RCT acoustic logging tool (Wuhan Zhongyan Technology Co., Ltd., Wuhan, China) was used to test the variation in compressional wave velocity due to different water content at different depths in the test site. The test data are shown in Table 1.

The test results show that the water content increases with the depth of the soil layer, and the velocity of the longitudinal wave increases accordingly. The test results are consistent with the conclusions of study [21]: the overall trend of water content and P-wave velocity in cohesive soil is positively correlated. When the soil moisture content is high, the P-wave velocity increases with the increase of water content within a certain range.

**Figure 1:**
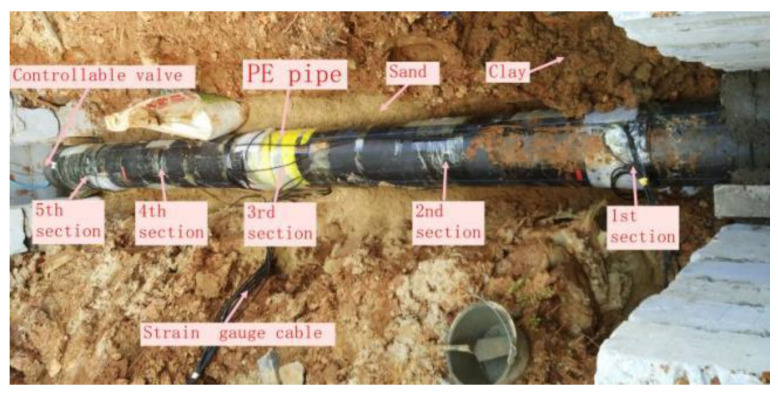
Test pipe diagram.

The test object is a PE80 water pipe, which is commonly used for oil and gas transmission in urban construction, as shown in Figure 1. PE pipe material parameters and geometric parameters are shown in Table 2 [22]. The pipeline laying method is a direct burial type laying, using professional machinery to excavate the pipe trench, to carry out the burial work in situ, using the original site soil backfill. Rock emulsion explosive was used in the blasting test, which was made into a spherical package and used coupled charging method.

The parameters changed in this experiment include the internal pressure of the pipe, the burst center distance, the buried depth of the burst source, and the amount of explosive. The experiments were designed using the control variable method and further optimized according to the orthogonal method, the test parameters and control variables range as shown in Table 3.

### 2.2. Test Monitoring System

**Figure 2:**
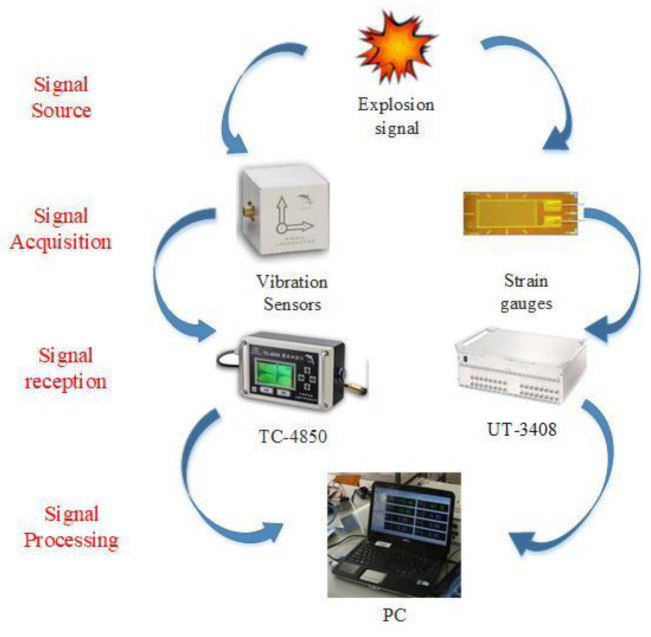
Schematic diagram of the monitoring system.

According to the test design plan, a dynamic tester was used to test buried pipe in the model test, and the relevant data were measured dynamically in real time during the blast test [14]. The main test items of the test included pipe dynamic strain, pipe vibration velocity, and ground surface vibration velocity above the pipe. The monitoring system of this experiment uses a TC-4850 blast vibrometer (China Chengdu Zhongke Measurement and Control Co., Ltd., Chengdu, China) and a UT-3408 dynamic strain tester (China Wuhan Youtai Electronic Technology Co., Ltd., Wuhan, China) to monitor the pipeline vibration and strain under the blast load in the test, and the monitoring system is shown in Figure 2.

#### 2.2.1. Vibration Test Systems

In order to study the vibration velocity of the buried pipeline and the surface above the pipeline, the TC-4850 blast vibration tester selected for the test was set up with 2 vibration velocity monitoring points on the upper surface of the pipeline and the surface of the soil above the pipeline, as needed. The TC-4850 vibrometer instrument used in the test has been calibrated regularly by a domestic professional evaluation agency. The sensor converts the velocity of the seismic wave generated by the explosion into a voltage signal, which is converted into a digital signal through a converter, and recorded into the memory of the instrument. After the test is completed, the data line is transferred to a computer, where the signal is calculated and processed, and finally output in the form of a report to a printer or stored on a hard disk drive. The main parameters of the vibrometer are shown in Table 4.

#### 2.2.2. Strain Testing Systems

The test strain is tested with a UT3408 dynamic strain tester; technical specifications are shown in Table 5, and professional components common in the BX120-3AA type resistance strain gauge (China Beijing Yiyang Vibration Testing Technology Co., Ltd., Beijing, China) and specific parameters are shown in Table 6.

**Figure 3:**
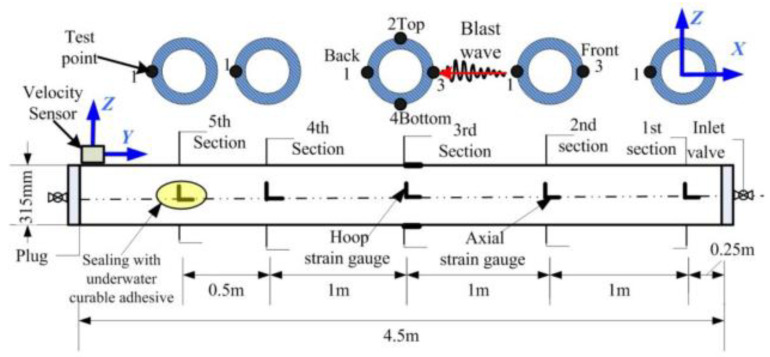
Pipeline strain and vibration test layout.

In order to study the dynamic strain of the pipeline during blast vibration, the dynamic strain measurement points of the pipeline are arranged as shown in Figure 3. Five cross-sections are selected on the outer surface wall of the pipeline, and four measurement points are arranged at the center of the pipeline, i.e., 3rd section, on the blast surface, back blast surface, top surface, and bottom surface, and one blast surface measurement point is arranged on the other sections. The strain gauges used in the test are uniaxial 80 mm, 120 ω strain gauges, which are wired to the UT3408 dynamic strain tester, which is suitable for all types of field tests by collecting physical quantities, such as stress–strain, voltage, displacement, charge, and acceleration.

### 2.3. Test Monitoring System

**Figure 4:**
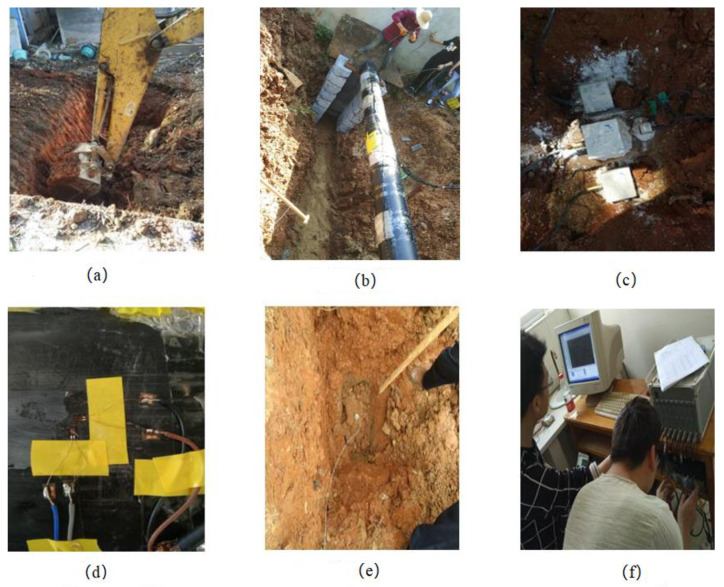
Schematic diagram of the test procedure; (**a**) Excavating trenches; (**b**) Buried pipelines; (**c**) Installation of vibration sensors; (**d**) Mounting strain gauges; (**e**) Placing explosives; (**f**) Data collection.

The implementation process of this experiment is shown in Figure 4.

#### 2.3.1. Pipe Sealing and Burial

Buried pipelines are mostly used for water, oil and gas transmission. In order to make the test results more realistic, this test simulates gas pipelines and pressurizes the PE pipes in the test. Therefore, it is necessary to add ends and valves at both ends of the pipe to connect air compressors to pressurize the inside of the pipe. At the thread contact surface of the pipe, pipe thread sealant was used to ensure sealing between the pipe ends and the valve. After it sets, close the valve on one side, pressurize it with an air compressor, and monitor its air tightness. If there is any air leakage, the above steps should be repeated until the pipe, end, and valve are all sealed. All thread contact surfaces are coated with pipe thread sealant to ensure the sealing of the entire structure.

#### 2.3.2. Strain Gauge Arrangement

The test method of strain testing in a general atmospheric environment is more mature, and it has the advantages of relatively easy operation and reliable results. However, the experimental pipeline was buried in watery soil, and the strain gauges were working in a watery environment, which might affect the strain gauges to a certain extent, and even cause damage to the strain gauges. In order to prevent damage to the strain gauges by immersion in the wet environment, the strain gauges were fully dried and cured, and the wires were connected to the strain gauges, then fixed with insulating glue after they were completely dry, and coated with water glue as a protective layer. After the water glue is dry, the wound wire is taped and fixed to prevent damage to the strain gauge when the wire is pulled.

As shown in Figure 3, five cross-sections were selected on the pipe, with four measurement points at the center of the pipe, i.e., 3rd section, on the blast face, back blast face, top face, and bottom face, and one blast face measurement point on the other sections. Two mutually perpendicular strain gauges in the axial and circumferential directions are arranged at each measurement point, the cable is tied and fastened to the center of the pipe, and the ends of the cable are marked according to the number of the measurement point, e.g., 3-1, i.e., measurement point 1 of 3rd section.

#### 2.3.3. Vibration Sensor Arrangement and Installation

Before installing the sensor, the ground and pipe surface are cleaned, and gypsum powder is used in the fastened ground and pipe to fix it, to ensure that the sensor and the ground and pipe between the connection to ensure the accuracy of the test. When installing the sensor on the pipe, the Y direction is parallel to the axial direction of the pipe, the X direction pointing to the source of the explosion, and the Z direction is vertically facing upward; when installing the sensor on the ground, the X direction is facing the source of the explosion and the Z direction vertically facing upward, while ensuring that the sensor is level. The sampling frequency selected for the experimental process testing are 8 KHz.

#### 2.3.4. Detonation and Testing

Before each test, according to the established charge design, carry out charge blockage, and perform extensive protection and alerting of people, to prevent harmful effects such as flying stones during the blasting process, and connect the charge to the detonator. Connect the data testing instrument with the sensor, and the relevant test personnel enter the experimental building to conduct test and test operations. According to the test plan, single or multiple blastholes are detonated, and data such as strain and vibration velocity are collected. After the single blasting, check the blasting effect, backfill around the blast hole, process the data, debug the test equipment, then carry out the next blasting test. The above steps are repeated to achieve the purpose of the experiment.

## 3. Test Results and Analysis

### 3.1. Dynamic Strain Analysis

Due to unavoidable factors in the test process, resulting in damage to some strain gauges, some data were missing or abnormal; after eliminating incomplete data strain signals, data were obtained for the burst source buried H = 1.5 m, burst center distance R = 2.7 m, 3.2 m, and 3.75 m, Q = 50 g, 75 g, 100 g, 125 g, 150 g, 175 g, and 200 g blast test conditions at each measurement point of the circumferential (H) peak strain and axial (Z) peak strain. The peak tensile strain (PTS) and peak compressive strain (PCS) were obtained, and the peak strain data of some pipes are shown in Table 7, Table 8 and Table 9.

**Figure 5:**
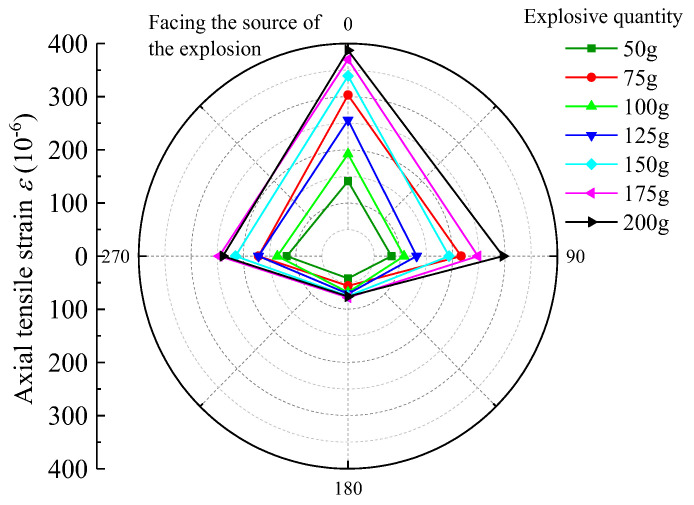
PE pipe 3rd section axial PTS distribution chart.

**Figure 6:**
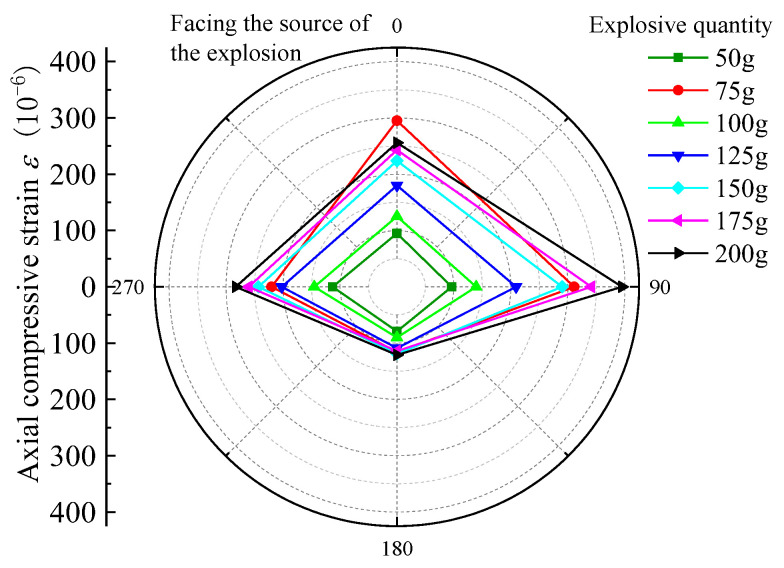
PE pipe 3rd section axial PCS distribution diagram.

**Figure 7:**
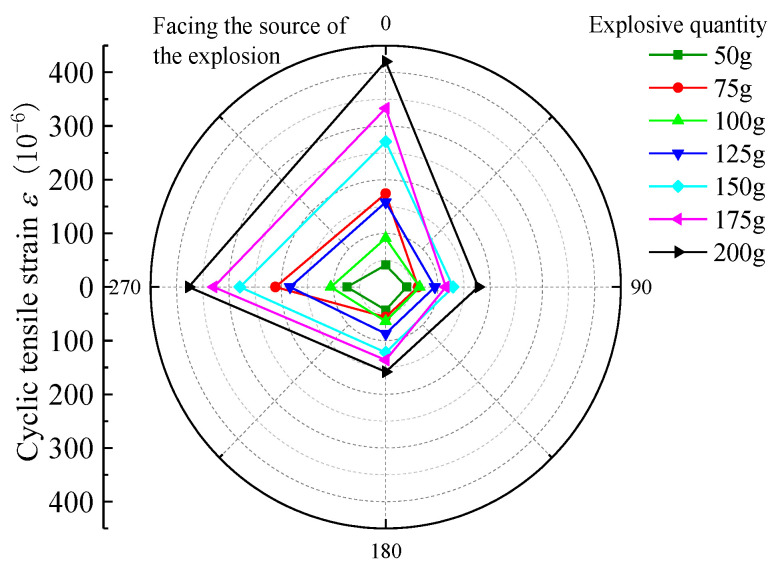
PE pipe 3rd section ring PTS distribution map.

**Figure 8:**
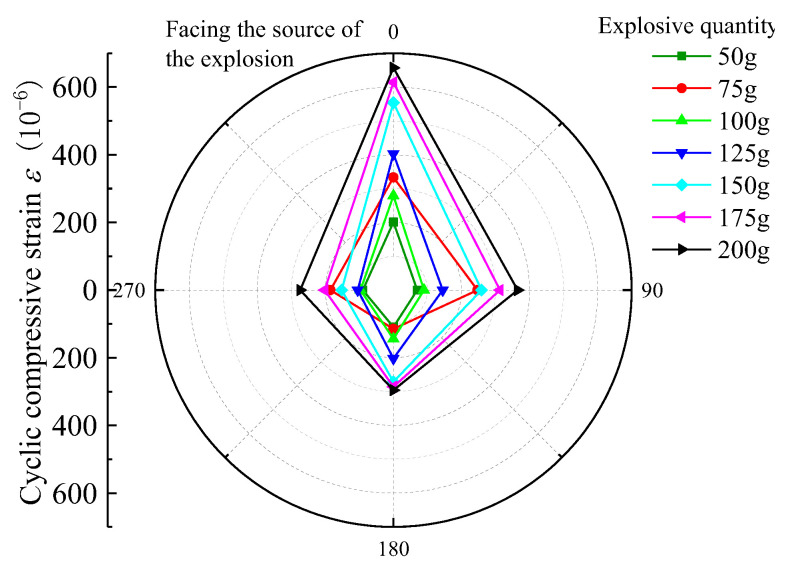
PE pipe 3rd section ring PCS distribution diagram.

For the nearest burst source of pipe to the center, that is, 3rd section, selected burst source burial depth H = 1.5 m, burst center distance R = 2.7 m, and Q = 50 g, 75 g, 100 g, 125 g, 150 g, 175 g, and 200 g strain data under each blasting test condition, the peak tensile strain (PTS) and peak compressive strain (PCS) distribution in the circumferential and axial directions are as follows in Figure 5, Figure 6, Figure 7 and Figure 8.

According to the above graphical data analysis, the peak strain of each measurement point of the pipe are increased with the increase in the charge can be seen. For 3rd section of the PE pipe (which is closest to the source), the maximum circumferential compressive strain is much greater than the tensile strain on the face (point 3) and back (point 1). The axial tensile strain is slightly larger than the compressive strain on the face of the explosion (measurement point 3). At the top and bottom of the pipe (measurement point 2 and measurement point 4), the circumferential tensile strain is generally larger than the compressive strain, and the axial compressive strain is generally larger than the tensile strain. Analysis shows that the outer surface of the pipe can be approximated as a plane stress state, the circumferential and axial strain is the main strain. Under different blasting conditions, the peak tensile and compressive strains on the back blast surface are within 45% to 57% of the peak on the blast surface. In the small scale distance, the maximum circumferential strain is larger than the maximum axial strain at the same measurement point. This is due to the fact that the test site is highly saturated clay, which has a high water content, which is conducive to the propagation of blast wave energy; the local impact of flexible PE pipe will produce in the larger circumferential strain. Based on the damage characteristics of polyethylene pipes, it can be seen that the pipes are more susceptible to damage under blast seismic loading, due to excessive circumferential compressive stresses.

### 3.2. Analysis of Vibration Test Results

Taking the burst center distance R = 2.7 m from the test data, calculations for the PE pipe and the surface PPV data as shown in Table 10. Here, PPV synthesis is by *x*-, *y*-, and *z*-axis maximum synthesis although, in general, the maximum value of the three does not occur at the same time, but the time difference is very small; synthesis of the highest value is slightly larger than the true highest value. The average ratio of the two is 0.386%, although the ratio of the two fluctuates with the proportional distance, but the fluctuation is not large in the range of the test proportional distance.

**Figure 9:**
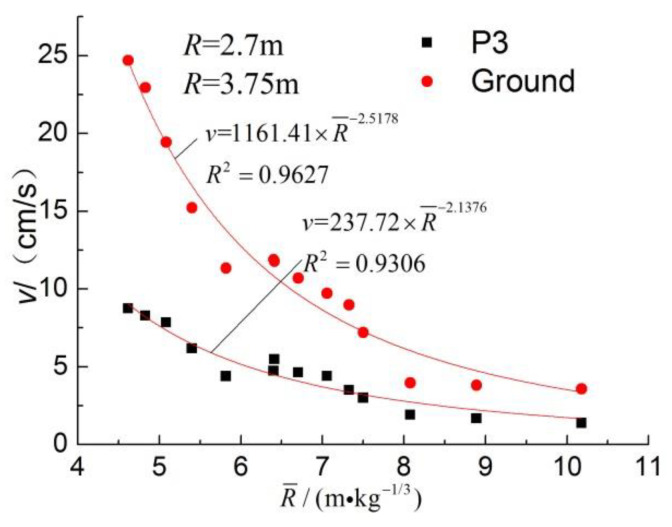
PPVV and GPPV decay with proportional distance.

**Figure 10:**
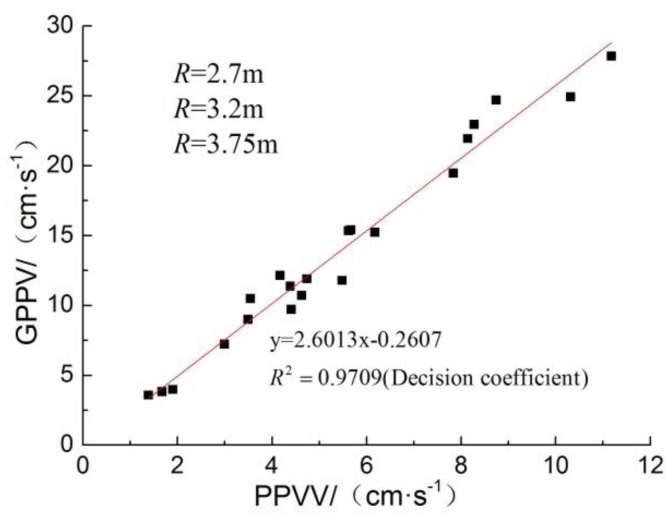
PPVV and GPPV linear correlation.

The two decay curves with proportional distance are shown in Figure 9, $\bar{R}$ $\left(m\cdot {\mathrm{kg}}^{-1/3}\right)$ represents proportional distance, $\bar{R}=R\cdot Q^{-1/3}$. The correlation between them is shown in Figure 10. PPVV and GPPV show a good power exponential function decay relationship with proportional distance, and the GPPV decay index is also larger than that in the related literature. There is a strong positive correlation between the pipeline and the inter-surface PPV, and the two have an approximately linear proportional relationship.

### 3.3. Peak Strain, GPPV, and PPVV Correlation Analyses

**Figure 11:**
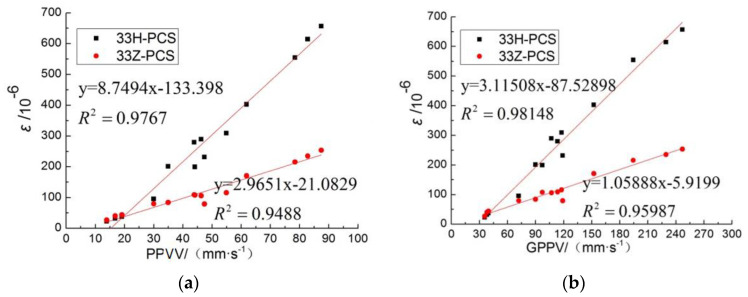
Peak strain is linearly related to PPPV (**a**) and GPPV (**b**) (R = 3.75 m, R = 2.7 m).

The results of the correlation between peak strain and PPV are shown in Figure 11. The peak strain at each measurement point is linearly correlated with the height of PPVV, and the PPPV is linearly correlated with the height of GPPV, which means that the peak strain is linearly correlated with the GPPV.

## 4. Safety Criterion for Bursting Vibration Power of Buried Pipelines

### 4.1. Pipe Stress Calculation

According to the strength calibration of the national standard pipeline, the axial and circumferential working stresses of the pipeline caused by the internal pressure and temperature changes at the maximum working pressure are calculated according to the following equation [23]:

(1) $$\begin{array}{l} \sigma_{hw}=\frac{pD_{out}}{2\delta }\\ \sigma_{aw}=\mu_{p}\sigma_{hw}+E_{p}\alpha_{l}\left(t_{1}-t_{2}\right) \end{array}$$

where *σ_h__w_* is the circumferential working stress at working pressure, *σ_α__ω_* is the axial working stress due to changes in working pressure and temperature, *α_l_* is the material linear expansion coefficient, *t*_1_ is the installation temperature, and *t*_2_ is the operation temperature.

In addition to the working stresses caused by the transport pressure, there are additional stresses caused by the blast load in the pipeline in service under the action of the blast wave. Additional stress and additional strain to perform conversion calculations can be considered the stress–strain principal structure relationship, to obtain appropriate simplification. According to the analysis of the test data, the strain in the 45 degree direction is small, and the circumferential and axial strains are the main strains, the pipeline vibration frequency is low, and the loading process can be treated as a quasi-static process. Ignoring the positive pressure on the surface of the pipe, the outer surface of the pipe can be simplified to a plane stress state, and the circumferential and axial stresses are the main stresses. The total circumferential and axial stresses on the surface of the pipe are calculated by the following formula:

(2) $$\begin{array}{l} \sigma_{h}=\sigma_{hw}^{}+\sigma_{h}^{\prime }\\ \sigma_{a}=\sigma_{aw}^{}+\sigma_{a}^{\prime } \end{array}$$

where $\sigma_{h}^{\prime }$ is additional circumferential stress and $\sigma_{a}^{\prime }$ is additional axial stress.

Since peak axial and circumferential strains do not always occur simultaneously, and both do not always have the same polarity (same tensile or same compressive), it is assumed that the stress–strain relationship follows a simplified generalized Hooke’s law in the inline elastic deformation range, as follows:

(3) $$\begin{array}{l} \varepsilon_{h}^{\prime }=\frac{1-\mu_{p}^{2}}{E_{p}}\sigma_{h}^{\prime }\\ \varepsilon_{a}^{\prime }=\frac{1-\mu_{p}^{2}}{E_{p}}\sigma_{a}^{\prime } \end{array}$$

where $\varepsilon_{h}^{\prime }$ is the additional circumferential strain and $\varepsilon_{a}^{\prime }$ is the additional axial strain;

The additional stress can be obtained from the experimental measured strain data by Equation (3).

### 4.2. Strength Conditions

The national standard for pipe strength calibration uses the maximum shear stress theory; however, the general plastic material is better to use the von Mises strength theory and engineering practice match. The strength theory is the distortion energy criterion; for PE pipes, the von Mises failure theory is used to calibrate the strength with the following calculation formula:

(4) $$\sigma =\sqrt{\frac{1}{2}\left[{\left(\sigma_{1}-\sigma_{2}\right)}^{2}+{\left(\sigma_{2}-\sigma_{3}\right)}^{2}+{\left(\sigma_{3}-\sigma_{1}\right)}^{2}\right]}\leq \sigma_{j}$$

where *σ*_1_, *σ*_2_, and *σ*_3_ are the first, second and third principal stresses of the three-way stress state, respectively, and *σ_j_* is the material’s ultimate stress.

The pipeline under the action of the blast wave, the most dangerous point on the outer surface of the pipeline in the most dangerous section, is simplified to a two-way stress state, considered in the most unfavorable case: assuming that the first and second principal stresses have the same sign and the third principal stress is zero, the von Mises failure theory can be simplified to the following inequality:

(5) $$\sqrt{\sigma_{1}{}^{2}+\sigma_{2}{}^{2}+\sigma_{1}\sigma_{2}}\leq \sigma_{j}$$

Equation (5)’s various materials ultimate stress selection is different. For the PE material pipeline’s ultimate stress, considering the importance of natural gas pipeline and with reference to the design of PE pipeline, the maximum working pressure (MOP) is mainly determined by the minimum required strength (MRS) of the material, so it is recommended that the persistence index MRS should be used as the final ultimate stress value of PE material, not the strength limit of PE material. MRS is the minimum guaranteed value of circumferential tensile strength of PE pipe for 50 years of normal operation under rated working pressure at 20 °C. MRS is much lower than the yield limit of polyethylene material, and the pipe stress is within the linear elasticity of the material, so this value is taken with a large safety margin. The national standard stipulates that the overall design factor of oil and gas transmission pipeline should be more than 2, so the rated pressure of polyethylene pipe has more than 30% safety margin in material strength. If the total time of blasting load on the pipeline is not long and the frequency of construction is not high, the accumulated damage can be negligible.

### 4.3. PE Pipe Safety Calibration

The disadvantage of the PE pipeline is the lower mechanical strength in higher working pressures and impact loads, which occur easily under the action of toughness fracture. In-service gas pipelines under the action of blast wave safety verification, by the internal pressure and shock load triggered by the combined stress judgment, according to the stress state calculated evaluation indicators, should be within the safety threshold, with strength verification according to the following formula:

(6) $$\sqrt{\sigma_{1}{}^{2}+\sigma_{2}{}^{2}+\sigma_{1}\sigma_{2}}\leq MRS$$

For the pipeline, additional annular and axial dynamic strain can be calculated according to the empirical formula fitted by field measurements, but also according to the practical calculation model of pipeline deformation established in this paper, with additional dynamic stress according to the Formula (3). In general, in the blast wave near the midfield pipeline annular stress and axial stress, (the first principal stress and the second principal stress, respectively), the plane stress state of the third principal stress is zero, Formula (2) brought into Formula (6) can obtain the following:

(7) $$\sqrt{{\left(\sigma_{hw}^{}+\sigma_{h}^{\prime }\right)}^{2}+{\left(\sigma_{aw}^{}+\sigma_{a}^{\prime }\right)}^{2}+\left(\sigma_{hw}^{}+\sigma_{h}^{\prime }\right)\left(\sigma_{aw}^{}+\sigma_{a}^{\prime }\right)}\leq MRS$$

In accordance with the PE pipe design regulations, the overall design factor CF must be greater than 2; here, a conservative value of C*_F_* equal to 2, the pressure reduction factor D*_F_* takes a value of 1, axial stress temporarily disregards the influence of temperature changes, and the joint vertical (5). Combining Formulas (6) and (7) to calculate the rated pressure of the pipe under the circumferential and axial working stress results in the following formula:

(8) $$\begin{array}{l} \sigma_{hw}=\frac{MRS\times D_{out}}{2\times \delta \times \left(SDR-1\right)}=\frac{MRS\times SDR}{2\left(SDR-1\right)}\approx \frac{MRS}{2}\\ \sigma_{aw}=\mu_{p}\sigma_{hw}\approx \frac{\mu_{p}\cdot MRS}{2} \end{array}$$

It can be seen that the circumferential working stress is about 50% MRS. Substituting Equation (8) into Equation (7) by trial calculation to obtain the maximum additional dynamic stress threshold under the rated working pressure of the pipeline PE pipe gives:

(9) $$\begin{array}{l} \sigma_{h}^{\prime }\leq 19\%MRS;\\ \sigma_{a}^{\prime }\leq 19\%MRS;\\ C_{F}=2 \end{array}$$

For the tested PE80 material pipe with MRS of 8 MPa, the test pipe was of the SDR17.6 series, with a maximum working pressure of 0.5 MPa and an annular working stress of 4 MPa. Because the relative stiffness coefficient of the pipe soil is relatively small, the annular stress, rather than the axial stress, becomes the main controlling factor. If the relative stiffness coefficient of pipe soil is larger, the additional axial stress may be the main controlling factor. The maximum value of additional dynamic stress for $\sigma_{h}^{\prime }$ should be 19% of MRS, which is about 1.5 MPa.

For conditions similar to this test, the peak strain of the pipe and the peak ground vibration velocity can be directly calculated using the fitted relationship equation. According to the recommended blasting safety guidelines, in this test site and test pipeline conditions, the PE pipeline pressurized to 0.5 MPa, according to the peak strain of the test data—the proportional distance fitting equation to calculate the maximum allowable explosives in the determination of the burst center distance. According to national regulations, natural gas pipelines within 5 m of blasting operations are prohibited, so take the burst core distance to be 5 m and the depth of burial of the package to be 1.5 m. For safety, monitoring of the maximum allowable GPPV can be fitted by peak strain and surface vibration speed relational equation, as shown in Figure 10; surface vibration speed and proportional distance relational equation is shown in Figure 11, the calculated parameters and results are shown in Table 11, and the fitted relational equations are as follows.

Cyclic peak strain versus GPPV:

(10) $$\varepsilon_{h\max}=31.2v_{l\max}-87.53$$

GPPV versus scaled distance:

(11) $$v_{l\max}=1161.41{\bar{R}}^{-2.5178}$$

The calculated maximum allowable GPPV of the PE pipe is 291 mm/s, which is much larger than the standard recommended in other literature. Under the test conditions, blast waves of this vibration amplitude did not pose a threat to the straight section of the PE pipe. After the completion of the test program plan, the final additional test, the pipeline pressurized to 0.6 MPa, greater than the maximum rated pressure of 0.5 MPa specified by the design. At a burst center distance of 2.2 m and a charge of 200 g, the peak surface vibration was greater than 291 mm/s, the maximum peak strain and fitting the results of the relationship between the calculation of a better match, the explosion did not occur after the pipe leakage and there was no pressure change in the pipe.

## 5. Conclusions

The bursting test of buried PE pipeline was carried out by using TC-4850 bursting vibrometer and UT-3408 dynamic strain tester to monitor the dynamic response of the pipeline. Combined with relevant theoretical analysis, the effect of blasting seismic load on a buried, pressured PE pipeline was studied. The primary conclusions are as follows:

(1) Through the field test found that the PE pipeline is affected by blasting vibration, the pipeline vibration speed and peak strain are increased with the increase in the amount of explosive, and the peak strain at the nearest location of the pipeline from the blast source is the largest. Among them, the PE pipe is subjected to the largest circumferential compression strain, and circumferential compression damage is more likely to occur in the pipe;
(2) According to the analysis of the test data, it can be seen that the peak vibration velocity of the pipeline, the peak surface vibration velocity, and the peak strain of the pipeline have a high linear correlation to the proportional distance of this test. The peak pipe strain and the peak surface vibration velocity satisfy the relationship $\varepsilon_{h\max}=31.2v_{l\max}-87.53$. It shows that the use of surface vibration velocity as a technical means of buried pipeline field monitoring is more reliable and simple to operate, and is worth promoting;
(3) Based on the test results, the safety evaluation of a buried, pressurized PE pipeline was carried out by combining the yield strength standard and relevant codes. The safety threshold of additional circumferential stress of pipeline in this experiment was 1.52 MPa, the safety control vibration speed of ground surface was 21.6 cm/s, the minimum value of safety proportional distance was 4.87 m kg^−1/3^, and the maximum allowable explosive quantity was 1.08 kg. The relevant conclusions can provide reference for the safety operation index of buried PE pipeline under similar working conditions.

## Figures and Tables

**Table 1:** Velocities of longitudinal waves in different depths of soil.

Serial Number	Sensor Type	Buried Depth/cm	Probe Distance/m	Longitudinal Sound Velocity/(m/s)
1	Acoustic emission probe	20	0.5	853
2	Aqueous medium coupling probe	60	0.5	891
3	Aqueous medium coupling probe	100	0.5	954

**Table 2:** PE pipe material parameters.

Outside Diameter of Pipe *D_N_*/mm	Inner Diameter of Pipe *d_s_*/mm	Wall Thickness of Pipe *δ_P_*/mm	Pipe Length *L_P_*/m	Young’s Modulus *E_P_*/MPa	Poisson’s Ratio *μ_p_*	Strength Limit *σ_P_*_b_/MPa	Elongation Rate *ξ_p_*/%
315	278	18.4	4.8	834.9	0.40	31.6	116

**Table 3:** Test parameters and range of control variables.

Experimental Parameters	Control Variable Range
Explosive quantity (g)	50, 75, 100, 125, 150, 175, 200
Depth of burial of explosion source (m)	0.5, 1, 1.5, 2
Pipeline internal pressure (MPa)	0, 0.2, 0.4, 0.6
Burst core distance (m)	2.2, 2.7, 3.2

**Table 4:** Blast vibration tester parameters.

Instrument:	TC-4850 Blast Vibrometer
Number of channels	Parallel three-channel
Display method	Full Chinese LCD display
Sampling rate	100 sps~100 Ksps, multi-grade adjustable
A/D resolution	16 Bit
Frequency response range	0~10 kHz
Recording method	Continuous trigger recording, can record 128~1000 times
Recording time	1~160 s adjustable
Trigger mode	Internal trigger, external trigger
Measurement range	Maximum input value 10 V (35 cm/s)
Trigger Level	0~10 V (0~35 cm/s) arbitrary adjustable
Storage Capacity	1M SRAM, 128 M flash
Recording accuracy	0.01 cm/s
Reading accuracy	1‰
Battery duration	≥60 h
Adaptation to the environment	−10~75 °C, 0~95% RH
Size	168 mm × 99 mm × 64 mm
Weight	1 kg

**Table 5:** Dynamic strain tester parameters.

Instrument:	UT-3408 Dynamic Strain Tester (Strain Measurement)
Number of channels	8/16, fully synchronized sampling
Maximum continuous sampling frequency	128 KHz
Dynamic range	120 dB
Input method	Each channel in the collector has built-in voltage, charge, IEPE (ICP), and strain input conditioning modules. Each channel can be independently programmed with voltage input, charge input (piezoelectric acceleration sensor), dual constant current source IEPE input (ICP acceleration sensor), and strain input, and each channel input mode can be programmed by software, and the indicator shows the setting status.
Programmable amplification	Automatic range control and pre-conditioning amplification (1, 2, 5, 10, 20, 50, 10, 200, 500, 1000×)
Strain measurement range	0~±100,000 με (2 V bridge voltage)
Bridge Circuit Resistor	60~1000 Ω
Bridge supply voltage (DC)	1, 2, 3, 6, 10, 12 V
Error difference	Errors ≤ ±0.2%
Current	20 mA max
Sensitivity factor	k = 2.00
Anti-interference energy	Can effectively resist 50 Hz interference

**Table 6:** Parameters of BX120-3AA type resistance strain gauge.

Resistance Value	Substrate Size	Wire Shed Size	Strain Rate Coefficient	Lead Length	Room Temperature Limit
120 ± 1 Ω	15 × 6 mm	3 × 3 mm	2.0 ± 1%	4 cm	20,000 UM/M

**Table 7:** PE pipe strain peak (R = 2.7 m).

Strain Gauges	Peak Strain/10^−6^													
	50 (g)		75 (g)		100 (g)		125 (g)		150 (g)		175 (g)		200 (g)	
	PTS	PCS	PTS	PCS	PTS	PCS	PTS	PCS	PTS	PCS	PTS	PCS	PTS	PCS
321H	46	121	86	151	73	160	99	239	143	318	162	340	182	356
321Z	42	79	56	116	68	90	71	109	75	119	78	114	76	121
323H	40	153	62	82	65	210	94	315	129	419	115	345	177	480
323Z	137	103	139	97	141	107	200	154	256	197	178	169	292	232
331H	43	109	56	115	64	144	87	202	122	271	136	286	158	296
331Z	95	84	62	79	113	109	137	171	159	216	164	235	159	254
332H	121	70	247	247	167	90	263	145	341	259	371	313	442	365
332Z	83	96	216	311	107	139	131	209	193	290	248	341	296	395
333H	41	201	174	333	91	279	158	403	271	554	333	614	420	657
333Z	141	95	303	295	192	125	256	180	339	224	370	243	387	256
334H	74	90	211	185	105	97	183	105	279	152	329	202	377	274
334Z	117	113	171	220	135	145	171	202	215	243	246	260	239	283

**Table 8:** P3 tube strain peak (R = 3.2 m).

Strain Gauges	Peak Strain/10^−6^													
	50 (g)		75 (g)		100 (g)		125 (g)		150 (g)		175 (g)		200 (g)	
	PTS	PCS	PTS	PCS	PTS	PCS	PTS	PCS	PTS	PCS	PTS	PCS	PTS	PCS
2-1H	119	162	83	114	100	121	117	178	198	274	154	244	178	264
2-1Z	109	120	80	99	86	116	98	135	142	176	120	162	123	173
2-3H	83	87	70	80	75	88	83	90	92	138	81	128	90	123
2-3Z	112	132	87	129	113	134	115	140	180	169	149	135	170	148
3-1H	94	104	72	70	87	76	108	93	170	182	147	115	164	143
3-1Z	69	87	53	72	58	77	69	80	95	99	78	77	79	87
3-2H	185	176	114	105	151	127	256	183	477	369	361	224	442	298
3-2Z	148	249	94	159	106	201	161	306	326	510	175	399	254	478
3-3H	133	284	70	187	93	205	163	316	287	502	210	385	245	436
3-3Z	219	228	139	153	167	187	243	264	429	397	300	279	357	328
3-4H	119	122	83	71	100	86	117	128	198	259	154	179	178	217
3-4Z	109	200	80	152	86	172	98	199	142	273	120	201	123	234

**Table 9:** P3 tube strain peak (R = 3.75 m).

Strain Gauges	Peak Strain/10^−6^													
	50 (g)		75 (g)		100 (g)		125 (g)		150 (g)		175 (g)		200 (g)	
	PTS	PCS	PTS	PCS	PTS	PCS	PTS	PCS	PTS	PCS	PTS	PCS	PTS	PCS
2-1H	21	21	23	24	25	27	39	67	51	128	51	141	68	181
2-1Z	20	19	28	24	32	27	48	50	57	61	50	47	60	58
2-3H	20	18	23	29	23	40	35	88	47	150	42	174	51	213
2-3Z	22	29	28	38	34	43	62	68	96	92	107	78	130	104
3-1H	20	20	24	26	26	28	41	53	52	102	49	120	66	147
3-1Z	13	23	21	31	27	35	47	67	68	95	62	84	82	110
3-1X	5	7	6	6	6	7	15	14	27	25	30	35	38	37
3-2H	16	11	26	16	29	19	69	41	134	88	194	110	195	135
3-2Z	18	24	24	37	24	41	42	76	65	104	76	122	88	122
3-3H	29	23	34	31	28	37	44	96	71	199	99	289	115	309
3-3Z	19	27	29	41	34	44	61	80	97	108	122	106	136	116
3-4H	18	18	22	28	24	32	43	51	99	69	155	55	165	65
3-4Z	19	30	26	41	30	43	51	84	82	121	88	114	103	139

**Table 10:** Comparison of GPPV and PE pipe PPVV (R = 2.7 m).

Projects	PPV (cm·s^−1^)						
	50 g	75 g	100 g	125 g	150 g	175 g	200 g
PPVV	3.5	4.7	4.4	6.2	7.8	8.3	8.7
GPPV	9.0	11.9	11.4	15.2	19.4	22.9	24.7
Ratio	38.9%	39.9%	38.7%	40.6%	40.3%	36.1%	35.4%

**Table 11:** Fitting relational calculation parameters and results (p = 0.5 MPa).

Pipe	Burst Core Distance/m	Material Ultimate Stress/MPa	Additional Circumferential Stress Threshold /MPa	Additional Peak Circumferential Strain/10^−6^	Surface Vibration Speed Threshold/(mm/s)	Permissible Surface Vibration Speed/(mm/s)	Minimum Safe Proportional Distance/(m kg^−1/3^)	Maximum Allowable Explosive Quantity/kg
PE	5	8 (MRS)	1.52	1435	43.2	21.6	4.87	1.08

## Data Availability

Not applicable.
